# Metabolic Reconstruction of *Setaria italica*: A Systems Biology Approach for Integrating Tissue-Specific Omics and Pathway Analysis of Bioenergy Grasses

**DOI:** 10.3389/fpls.2016.01138

**Published:** 2016-08-10

**Authors:** Cristiana G. de Oliveira Dal'Molin, Camila Orellana, Leigh Gebbie, Jennifer Steen, Mark P. Hodson, Panagiotis Chrysanthopoulos, Manuel R. Plan, Richard McQualter, Robin W. Palfreyman, Lars K. Nielsen

**Affiliations:** ^1^Centre for Systems and Synthetic Biology, Australian Institute for Bioengineering and Nanotechnology, The University of QueenslandBrisbane, QLD, Australia; ^2^Metabolomics Australia, Australian Institute for Bioengineering and Nanotechnology, The University of QueenslandBrisbane, QLD, Australia

**Keywords:** *Setaria italica*, metabolic reconstruction, omics, plant systems biology, C4 photosynthesis, bioenergy grasses

## Abstract

The urgent need for major gains in industrial crops productivity and in biofuel production from bioenergy grasses have reinforced attention on understanding C_4_ photosynthesis. Systems biology studies of C_4_ model plants may reveal important features of C_4_ metabolism. Here we chose foxtail millet (*Setaria italica)*, as a C_4_ model plant and developed protocols to perform systems biology studies. As part of the systems approach, we have developed and used a genome-scale metabolic reconstruction in combination with the use of multi-omics technologies to gain more insights into the metabolism of *S. italica*. mRNA, protein, and metabolite abundances, were measured in mature and immature stem/leaf phytomers, and the multi-omics data were integrated into the metabolic reconstruction framework to capture key metabolic features in different developmental stages of the plant. RNA-Seq reads were mapped to the *S. italica* resulting for 83% coverage of the protein coding genes of *S. italica*. Besides revealing similarities and differences in central metabolism of mature and immature tissues, transcriptome analysis indicates significant gene expression of two malic enzyme isoforms (NADP- ME and NAD-ME). Although much greater expression levels of NADP-ME genes are observed and confirmed by the correspondent protein abundances in the samples, the expression of multiple genes combined to the significant abundance of metabolites that participates in C_4_ metabolism of NAD-ME and NADP-ME subtypes suggest that *S. italica* may use mixed decarboxylation modes of C_4_ photosynthetic pathways under different plant developmental stages. The overall analysis also indicates different levels of regulation in mature and immature tissues in carbon fixation, glycolysis, TCA cycle, amino acids, fatty acids, lignin, and cellulose syntheses. Altogether, the multi-omics analysis reveals different biological entities and their interrelation and regulation over plant development. With this study, we demonstrated that this systems approach is powerful enough to complement the functional metabolic annotation of bioenergy grasses.

## Introduction

### Need for a C_4_ model system

C_4_ photosynthesis drives productivity in several major food crops and bioenergy grasses, including corn, sugarcane, sorghum, and switchgrass (Sage and Zhu, 2011). Gains in productivity associated with C_4_ photosynthesis include improved water, carbon, and nitrogen use efficiencies. Therefore, understanding C_4_ metabolism and its underling regulatory network is fundamental for improvement of important industrial crops.

Over recent years, *Setaria italica* and its wild ancestor *Setaria viridis* (green millet); members of the Panicoideae clade and closely related to several of the major C_4_ bioenergy grasses (Defelice, 2002), have been proposed as the potential new models to fill this need for a C_4_ model plant (Doust et al., 2009; Brutnell et al., 2010; Li and Brutnell, 2011). Setaria model plants present a few advantages for physiological studies over bioenergy grasses such as: (i) relatively small genome (~450 Mb), (ii) simple growth requirements, and (iii) rapid life cycle (Doust et al., 2009; Brutnell et al., 2010; Li and Brutnell, 2011; Bennetzen et al., 2012; Zhang et al., 2012; Martins et al., 2015). Therefore, both Setaria species are likely to facilitate systems biology studies in order to understand C_4_ metabolism and its underling regulatory network.

### Genome-scale reconstruction and systems biology studies for model organisms

By having an experimental platform (plant model), the generated experimental data (wet side) can be integrated into an *in silico* platform (dry side) for systems biology studies. The *in silico* platform can be created through the characterization of entire networks (so called genome-scale metabolic reconstructions). This systems approach has enabled insights into biological processes revealing emergent properties of the biological networks (Resendis-Antonio et al., 2007; Oberhardt et al., 2009; Saha et al., 2014). A metabolic reconstruction is a well-structured description of the network topology that enables derivation of genome-scale models (GEMs) that are used to mimic different metabolic states of an organism (Satish Kumar et al., 2007; Thiele and Palsson, 2010). Such technology has gained popularity for systems biology studies as it enables the integration of omics and overall analysis to explore the interplay of metabolic networks (Saha et al., 2014). A few metabolic reconstructions have been developed for different plant species, including Arabidopsis (Poolman et al., 2009; de Oliveira Dal'Molin et al., 2010a; Mintz-Oron et al., 2012), maize (de Oliveira Dal'Molin et al., 2010b; Saha et al., 2011), sugarcane, and sorghum (de Oliveira Dal'Molin et al., 2010b). Although, challenges arise due to large genome sizes and metabolic complexity (de Oliveira Dal'Molin and Nielsen, 2013), the derived GEMs predicted important physiological scenarios (de Oliveira Dal'Molin et al., 2010a,b), including cooperative C_4_ photosynthesis in bundle sheath and mesophyll cells (de Oliveira Dal'Molin et al., 2010b), diurnal cycle in C_3_ and crassulacean acid metabolism in leaves (Cheung et al., 2014) nitrogen availability in maize leaf (Simons et al., 2014). More recently a multi-tissue genome-scale model framework was developed and used to investigate diurnal cycle and C/N translocation efficiency across the whole plant (de Oliveira Dal'Molin et al., 2015). In other recent study, multi-omics integration and modeling was used to elucidate the light-specific transcriptional signatures of rice metabolism (Lakshmanan et al., 2015). The reconstruction-modeling approach has proven a powerful tool to study the complexity of metabolism and is believed to advance plant metabolic engineering studies when used in combination with experimental design (de Oliveira Dal'Molin and Nielsen, 2013; de Oliveira Dal'Molin et al., 2014). Considering its potential use, a metabolic reconstruction of the Setaria model plant is likely to facilitate multi-omics integration and analysis in order to understand the interplay of metabolic networks in C_4_ plant metabolism. In this work, we developed a metabolic reconstruction of *S. italica* to perform systems biology studies. We have also developed protocols to perform omics analysis in mature and immature tissues. By implementing such an approach, we have attempted to capture key metabolic features in different developmental stages of the C_4_ model plant.

## Materials and methods

### Plant material

*S. italica* seeds were sown into grade 2 vermiculite (Ausperl) supplemented with Osmocote® (Scotts Australia) in a 6 × 5 well-plastic seedling tray. The seedling tray was placed in a 4 cm deep tray containing 3 cm of water and placed in a plant growth cabinet (Percival E41-HO) with a 12 light (28°C), 12 dark (24°C) cycle, with a light intensity of ~500 μmol/m^2^/s. The seedling tray was covered with a transparent plastic cover to retain moisture until seedlings emerged. The plants were watered regularly to maintain water in the bottom tray. Once per week the plants were fertilized with 100 mg/L of soluble fertilizer (Flowfeed Ex7, Grow Force). As seedlings emerged, they were thinned to four seedlings per well and grown until 35 days old at which point the plants contained both developing and mature stem/leaf phytomers. A mature phytomer was chosen from near the base of each plant, which contained a healthy, fully mature green leaf and leaf sheath, and the associated node together with the stem material above the node until just below the next node. As it is difficult to successfully isolate an intact individual, immature leaf/stem phytomer from the top of the plant, the whole top of the plant was removed above the node associated with the first fully unfurled leaf. Samples were pooled in groups of four to increase biomass. A total of 5 replicates of mature and immature pooled leaf samples were taken and snap frozen in liquid nitrogen. The plant material was ground to a fine powder in liquid nitrogen and stored at −80°C until required for analysis.

### Transcriptome

#### RNA extraction and sequencing

Total RNA was extracted from 100 mg of tissue using the Bio-Rad Aurum Total RNA kit for fatty and fibrous tissue as per the manufacturer's instructions, including the on-column DNaseI treatment. The quality of the RNA was determined using the Agilent Bioanalyser before proceeding (RIN = 7.6–8.7; data not shown). RNA sequencing, including library preparation, was performed by the genomics facility at the Kinghorn Centre for Clinical Genomics (Garvan Institute of Medical Research, 384 Victoria St, Darlinghurst, NSW 2010, Australia). Briefly, RNA-Seq libraries were prepared using the Illumina TruSeq Stranded mRNA Library kit using the standard protocol to produce libraries with an average size of 309 bp. These libraries were then sequenced on an Illumina HiSeq 2500 system (HiSEQ Control Software 2.2.38, RTA 1.18.61) following the standard rapid sequencing workflow. Samples were loaded at a concentration of 14 pM and a total of 209 cycles of sequencing were completed consisting of 2 × 101 bp reads and a single 7 bp index sequence.

#### Data processing

RNA-Seq reads were mapped to the *S. italica (Sitalica_164 from Phytozome v9.0)* reference genome using Tophat v2.0.12 (https://ccb.jhu.edu/software/tophat/; Kim et al., 2013), resulting in 78% of the total 350 million reads mapping. Differential expression was determined using Cufflinks v2.2.1 (http://cole-trapnell-lab.github.io/cufflinks/; Trapnell et al., 2013). Each of the six BAM files (three per condition) generated by Tophat2 were converted to abundance files using cuffquant. The two sets of tripilicates were then compared with cuffdiff using geometric normalization and with fragment bias correction and multi read correction. The annotation file used was the Phytozome v9.0 Sitalica_164_gene.SETIT gff3 file.

#### Gene ontology

Gene ontology analysis was performed using agriGO: A web-based database for gene ontology analysis, that supports special focus on agriculture species (Du et al., 2010).

### Proteome

#### Protein extraction

The proteome of four biological replicates of mature and immature stem/leaf phytomers were analyzed. Proteins were extracted by lysing 100 mg of chopped mature or immature tissues with 2% SDS, 6.4 M urea, 0.1 M Tris-HCl, and 0.1 M dithiothreitol buffer at pH 8.5. Therefore zirconia beads (0.5 mm diameter) were added to the samples and homogenized (Precellys 24, Bertin) using a liquid nitrogen cooler (Cryolys, Bertin). The homogeniser operated at 11°C with 3 cycles of 45 s at 6500 rpm, with 30 s intervals between cycles. Samples were then centrifuged at 13,000 rpm for 10 min at 4°C, and the protein concentration in the supernatant was measured using the 2D Quant Kit (GE Healthcare).

#### Protein digestion

Samples were digested using filter aided sample preparation with some modifications (Wisniewski et al., 2009; Abdallah et al., 2012). Briefly, 150 μg of protein was diluted to a total volume of 200 μL with buffer A (8 M urea in 0.1 M Tris–HCl, pH 8.5), loaded into Amicon Ultra-0.5 mL centrifugal filters with nominal cutoff of 30 kDa (Wisniewski et al., 2011; Millipore), and centrifuged at 14,000 × g for 15 min. Proteins were washed with 200 μL of buffer A by centrifugation at 14,000 × g for 15 min. Hundred microliters of 50 mM iodoacetamide in solution A was added and incubated in the dark at room temperature (RT) for 30 min. Samples were centrifuged at 14,000 × g for 15 min followed by two washes with 100 μL of buffer A. An additional wash with 100 μL of triethylammonium bicarbonate (TEAB) solution was performed. In-filter-digestion was undertaken overnight at 37°C with gentle agitation (50 rpm) using a trypsin to protein ratio of 1:10. Peptides were collected by centrifugation of the filter units at 14,000 × g for 15 min, followed by one additional 30 μL elution with TEAB.

#### iTRAQ labeling

Samples were labeled according to iTRAQ manufacturer's protocol. In summary, 50 μg of peptides from each sample was labeled with a different 8-plex iTRAQ reagent, switching between conditions, and incubated at room temperature for 2 h. Samples were then combined, concentrated using a vacuum centrifuge, and desalted using a Sep-Pak tC_18_ 1 cc Vac Cartridge (Waters). Acetonitrile from the elution buffer was removed with a vacuum centrifuge prior to fractionation.

#### Fractionation

The iTRAQ mixture was fractionated using an Agilent OFFGEL 3100 fractionator with a 24 cm GE Healthcare Immobiline DryStrip with a nonlinear pH range of 3–10. The strip was rehydrated with a buffer containing 4.8% glycerol and 0.96% IPG buffer pH 3–10 (GE Healthcare) for 15 min. Peptides were diluted in 3.6 mL of the buffer and added in equal amounts to each well. Fractionation was achieved using the default program for peptides “OG24PE01” which targets 50 kVh with maximum values of 4500 V, 200 mW, and 50 mA. The 24 resulting fractions were combined into 18 fractions and desalted using a Sep-Pak tC_18_ 1 cc Vac Cartridge (Waters). Each fraction was concentrated using a vacuum centrifuge and resuspended in 0.1% formic acid. Four microgram of labeled peptides were injected into the LC-MS/MS ((liquid chromatography tandem mass spectrometry).

#### LC-MS/MS analysis

Samples were separated on a Shimadzu Prominence nanoLC system as described elsewhere (Kappler and Nouwens, 2013), with the subsequent modifications. Peptides were desalted with an Agilent C18 trap (0.3 × 5 mm, 5 μm) at a flow rate of 30 μL/min for 3 min and separated on a Vydac Everest C18 (300 A, 5 μm, 150 mm × 150 μm) column at a flow rate of 1 μL/min, using a gradient of 10–60% buffer B1 over 75 min (buffer A1 = 1% ACN/0.1% formic acid and buffer B1 = 80% ACN/0.1% formic acid). Eluted peptides were immediately analyzed on a Triple-TOF 5600 instrument (ABSciex) equipped with a Nanospray III interface. Gas 1 was set to 10 psi, curtain gas to 30 psi, and ion spray floating voltage to 2700 V. Samples were scanned across *m/z* 350–1800 for 0.5 s followed by information-dependent acquisition on high sensitivity mode of 20 peptides with intensity >100 counts across *m/z* 40–1800 for 0.05 s. Rolling collision energy was used.

#### Data analysis

MS/MS data was analyzed using the Paragon Algorithm from ProteinPilot v4.5 (ABSciex, Forster City CA; Shilov et al., 2007) The 24 fractions were analyzed simultaneously using ProteinPilot. Protein sequences for *S. Italica* were downloaded from Phytozome v9.0 database, containing 34,725 proteins. Search parameters included false discovery rate analysis and “thorough” settings. Only proteins identified with at least 2 peptides with more than 95% confidence score and an unused score higher than the 5% local false discovery rate score of all identified proteins were included in the statistical analysis.

#### Statistical analysis

The protein ratios obtained by ProteinPilot were log_2_-transformed and fitted for each protein to a linear model using the R package Limma (Smyth, 2005). A moderated t-statistic test was calculated for the contrast using Limma's Bayes method, and if the adjusted *p* < 0.05, proteins were classified as differentially abundant in mature and immature tissues (Smyth, 2004).

### Targeted metabolome

#### Metabolite extraction

Metabolites were extracted for subsequent liquid chromatography using a modification from Glassop et al. (2007). Briefly, 100 mg frozen leaf powder was added to 700 μL extraction solution. The extraction solution consisted of 70 mL methanol, 200 μL of 10 mM ^13^C_5_^15^N-valine (aq), 200 μL of 1 mM ^13^C_6_-sorbitol (in MeOH), 200 μL of 5 mM 1,2-^13^C_2_-myristic acid (in CHCl_3_), 4 mL of 0.2 mg/mL adonitol + 0.2 mg/mL norleucine (aq) and 6 mL of 2 mg/mL nonadecanoate methyl ester (in CHCl_3_). Samples were immediately incubated at 70°C for 10 min with frequent inversion. Five hundred and eighty microliters of deionized water and 500 μL of CHCl3 were then added, and the sample was vortexed for 1 min. Polar and nonpolar phases were separated by centrifugation at 15,000 g for 10 min at 4°C. The polar phase was re-extracted with CHCl_3_.

#### Central carbon metabolites (LC-MSMS)

Reference standards and tributylamine (puriss plus grade) were purchased from Sigma Aldrich (Sigma Aldrich, NSW, Australia). HPLC Grade acetonitrile and acetic acid (AR Grade) was purchased from RCI Labscan (Bangkok, Thailand) and Labscan (Gliwice, Poland), respectively. Deionised water was generated via an Elga Purelab Classic water purification unit (Veolia Water Solutions and Technologies, Saint Maurice Cedex, France).

Liquid chromatography tandem mass spectrometry (LC-MS/MS) data were acquired on a Dionex UltiMate 3000 liquid chromatography system (Dionex, California, USA) coupled to an ABSciex 4000 QTRAP mass spectrometer (ABSciex, Concord, Canada). The liquid chromatography system was controlled by Chromeleon software (Dionex), and chromatographic separation was achieved by injecting 10 μL onto a Gemini-NX C18 150 × 2 mm I.D., 3 μm 110 Å particle column (Phenomenex, Aschaffenburg, Germany) equipped with a pre-column Security Guard Gemini-NX C18 4 × 2 mm I.D. cartridge. The column oven temperature was controlled and maintained at 55°C throughout the acquisition and the mobile phases (adapted from Luo et al., 2007) were as follows: 7.5 mM aqueous tributylamine adjusted to pH 4.95 (±0.05) with glacial acetic acid (eluent A) and acetonitrile (eluent B). The mobile phase flow rate was maintained at 300 μL/min throughout the gradient profile (see Table S1) and was introduced directly into the mass spectrometer with no split.

The mass spectrometer was controlled by Analyst 1.5.2 software (ABSciex) and was equipped with a TurboV electrospray source operated in negative ion mode. The following optimized parameters were used to acquire scheduled Multiple Reaction Monitoring (MRM) data: Ionspray voltage −4500 V, nebulizer (GS1), auxiliary (GS2), curtain (CUR), and collision (CAD) gases were 60, 60, 20, and medium (arbitrary units), respectively, generated via a N300DR nitrogen generator (Peak Scientific, Massachusetts, USA). The auxiliary gas temperature was maintained at 350°C. The analyte-dependent parameters for the detection of central carbon metabolites are shown in Table S2. For all analytes the entrance potential (EP) was −10 volts.

The samples were run with sample- and analyte-relevant calibration standards and pooled QC samples (Sangster et al., 2006; Hodson et al., 2009) to control for reproducibility of data acquisition and to ensure data integrity. Analyte stock solutions were prepared in purified water (Veolia) and aliquots of each solution were mixed to achieve a final calibrant solution at 200 μM. This calibrant solution was serially diluted and the dilutions used as calibration standards from 200 to 0.006 μM, constituting 7 ≤ *x* ≤ 20 calibration points to account for differential responses in the mass spectrometer. As an internal standard, 1 μl of a 1 mM aqueous solution of azidothymidine was added to 99 μl of sample. Data were processed using MultiQuant 2.1 software (AB Sciex).

#### Amino acid analysis

Amino acids were quantified using a high-throughput method developed from our previous work (Dietmair et al., 2010; Chacko et al., 2014). In brief, samples were diluted 1:1 with internal standards and derivatised amino acids were analyzed by RP-HPLC. Derivatisation was performed in a high-performance autosampler (Agilent HiP-ALS SL, G1367C). 0.5 μL of sample containing 250 μM of internal standards, sarcosine and 2-aminobutanoic acid, was added into 2.5 μL of borate buffer (0.4 N, pH 10.2, Agilent PN: 5061-3339), mixed and incubated for 20 s at 4°C. One microliter of OPA reagent (10 mg *o*-pthalaldehyde/mL in 3-mercaptopropionic acid, Agilent PN: 5061-3335) was then added to initially derivatise primary amino acids. The reaction was mixed and incubated for 20 s at 4°C. Then 0.4 μL of FMOC reagent (2.5 mg 9-fluorenylmethyl chloroformate/mL in acetonitrile, Agilent PN:5061-3337) was added, mixed, and incubated for 20 s at 4°C, to derivatised other amino acids. 45.6 μL of Buffer A (40 mM Na_2_HPO_4_, 0.02% NaN_3_, pH 7.8) was added to lower the pH of the reaction prior to injecting the 50 uL reaction onto an Agilent Zorbax Extend C-18 column (3.5 μm, 4.6 × 150 mm, Agilent PN: 763953-902) with a guard column (SecurityGuard Gemini C18, Phenomenex PN: AJO-7597). Column temperature was maintained at 37°C in a thermostatted column compartment (Agilent TCC, G1316B). Chromatography was performed using an Agilent 1200-SL HPLC system, equipped with an active seal wash and a degasser (Agilent Degasser, G1379B). The HPLC gradient was 2–45% B2 from 0 to 18 min, 50–60% B2 from 18.1 to 20 min, 100% B from 20.1 to 24 min, and 2% B2 from 24.1 to 27 min—using a binary pump (Agilent Bin Pump SL, G1312B). Buffer B was 45% acetonitrile, 45% methanol, and 10% water. Flow rate was 2 mL/min. Derivatised amino acids were monitored using a fluorescence detector (Agilent FLD, G1321A). OPA-derivatised amino acids were detected at 340_ex_ and 450_em_ nm from 1 to 18 min, and FMOC-derivatised amino acids at 266_ex_ and 305_em_ nm from 18 to 27 min. Chromatograms were integrated using ChemStation (Rev B.03.02[341]).

#### Sugars, alcohol, and organic acid analysis

Organic acids, sugars, and alcohol were quantified by ion-exclusion chromatography using an Agilent 1200 HPLC system and an Agilent Hiplex H column (300 × 7.7 mm, PL1170-6830) with guard column (SecurityGuard Carbo-H, Phenomenex PN: AJO-4490; McQualter et al., 2016). Sugars and alcohols were monitored using a refractive index detector (Agilent RID, G1362A) set on positive polarity and optical unit temperature of 40°C, while organic acids were monitored at 210 nm (Agilent MWD, G1365B). Thirty microliters of sample was injected onto the column using an autosampler (Agilent HiP-ALS, G1367B) and column temperature kept at 65°C using a thermostatted column compartment (Agilent TCC, G1316A). Analytes were eluted isocratically with 4 mM H_2_SO_4_ at 0.6 mL/min for 26 min. (Add the following sentence if applicable). To avoid high temperature acid hydrolysis, sucrose was analyzed separately at a column temperature of 15°C and by using high purity water (18.2 MΩ cm) as the mobile phase, and eluted isocratically at 0.4 mL/min for 21 min. Chromatograms were integrated using ChemStation (Rev B.03.02[341]).

#### GC-MS FAME analysis

Fatty acids were converted to their methyl esters and measured using a method based on published methods developed for GC-MS by Metabolomics Australia. Briefly, lipid extracts were saponified for 2 h at 80°C with 200 μL of 2 M NaOH and 400 μL of methanol. Upon acidification with 40 μL of 37.5% concentrated HCl, 400 μL of chloroform were added and the mixture vortexed thoroughly. Phase separation was accelerated by centrifugation for 3 min at 3000 × g. The chloroform layer was collected and evaporated in a vacuum centrifuge. Two hundred microliters of 2% H_2_SO_4_ in methanol were then added to the extracts and incubated for 2 h at 80°C. Once they reached RT, 200 μL of 0.9% NaCl were added and vortexed thoroughly. For the recovery of the fatty acid methyl esters (FAME) 300 μL of hexane were used. A volume of 2 μL of the hexane layer was injected directly in the GC-MS in splitless mode, at 350°C using helium as a carrier gas under a constant flow of 1 mL/min. Metabolites were separated on a Varian capillary column (Factor FOUR VF-5 ms: 0.25 mm i.d., 0.25 μm film, 30 m length with a 10 m fused guard column; Varian, Mulgrave, Australia) installed on an Agilent 7890A gas chromatograph coupled to an Agilent 5975C MSD mass spectrometer (Agilent Technologies, Santa Clara, USA). The initial temperature of the separation program (70°C) was held for 5 min, then increased to 320°C at a rate of 9°C/min and finally increased to 325°C at a 30°C/min rate and held for 6.3 min. The ion source, quadrupole, and transfer line temperatures were set at 300, 150, and 280°C respectively. The methyl esters of the fatty acids were identified by direct comparison with standard solutions and were processed in total ion count mode (TIC).

GC-MS metabolite peak identification was based on (a) an in-house library of standards and (b) on the commercially available NIST MS library (2012) with a match threshold of 70%.

Pre-processing of GC-MS data was performed using AMDIS (Automated Mass Spectral Deconvolution and Identification System version 2.65) software for peak de-convolution and peak integration. The deconvolution parameters were selected as follows: Component width = 6; adjacent peak subtraction = 2; resolution = medium; sensitivity = medium; shape requirement = medium. The data extracted from AMDIS were further processed using MassHunter Quantitative Analysis software for peak curation (version B.06.00, Agilent Technologies) and Mass Profiler Professional software (version 12.1, Agilent Technologies). All data were normalized to the internal standard (IS) intensity and were aligned with a retention time tolerance of 0.1 min. Once processed the data matrix was exported in.csv format for external data analysis.

### Metabolic reconstruction

The metabolic reconstruction of *S. italica* was developed based on its mRNA transcripts homologous mapping, using C4GEM (de Oliveira Dal'Molin et al., 2010b): a genome-scale model framework developed for the *in silico* analysis of C_4_ plant species. The plant metabolic reconstruction process is described in detailed in our previous works (de Oliveira Dal'Molin et al., 2010a,b). Briefly, the *S. italica* reconstruction consisted of a few steps:

- Firstly, we used C4GEM (framework developed *in house*) as a C_4_ metabolic core model, which holds primary metabolic functions shared among C_4_ plants. This framework adopts a gene-centric organization of metabolic information, in which each known metabolic gene is mapped to one or several reactions.
- Secondly, the *S*. *italica* genes were mapped to C4GEM by using BLAST to identify *Zea mays* genes in C4GEM. The set of unique reactions ID were extracted and stored as a stoichiometric matrix (Java application). In this step, multiple entries for a reaction in a particular compartment appearing in the Excel gene-enzyme-reaction table are collapsed to a single reaction entry.
- Finally, omics datasets of mature and immature tissues of *S. italica* were mapped to the metabolic reconstruction for functional pathway analysis.

## Results and discussion

### GO term analysis

The goal of functional profiling is to determine which processes might be different in particular sets of genes, a process that is often conducted by determining which Gene Ontology (GO) terms are differentially represented. The GO terms are organized in three general categories: Biological process, molecular function, and cellular component and the terms within each category are linked in defined parent-child relationships that reflect current biological knowledge (Gene Ontology, 2001).

**Figure 1:**
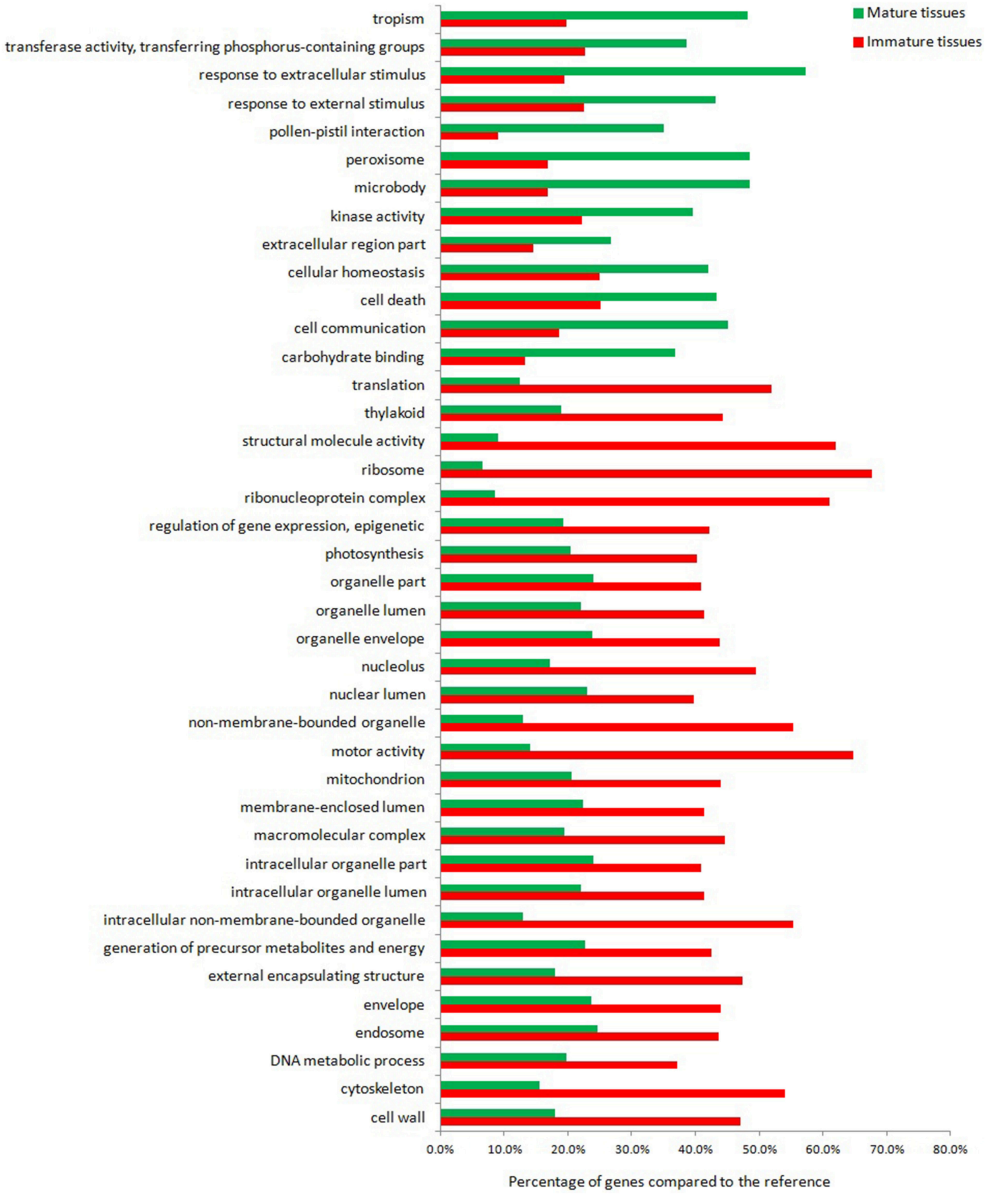
**Gene ontology of the most differentially expressed transcripts in mature and immature tissues of ***Setaria italica*****.

The result of our high-throughput experiment is a set of genes that are differentially expressed between mature and immature leaf/stem phytomers of *S.italica* (Figure 1). When performing such an analysis, two types of questions may be addressed: A hypothesis-generating query (such as which GO terms are significant in a particular set of genes) and a hypothesis-driven query (such as whether the response to external stimulus or photosynthesis is significantly enriched or depleted in a particular set of genes). Considering the most differentially expressed genes between mature and immature tissues, 40 major functional categories were identified. Approximately 70% of the presented categories represent transcripts with greater expression in immature tissues, whereas the remaining categories represent transcripts with greater expression in mature tissues. Overall, the GO term analysis indicates that the transcripts assigned to protein synthesis and cellular metabolic processes across different organelles to build biomolecules are up-regulated in young tissues, while genes assigned to catabolic and degradation pathways, homeostasis, abiotic stress, and cell death regulatory response are up regulated in mature tissues. A more comprehensive analysis toward the metabolic pathways context was adopted using omics data integrated to a genome scale metabolic reconstruction for further biological interpretation.

### *Setaria italica* metabolic reconstruction and multiple omics mapping

Interpretation of multi-omics results is a non-trivial task. Statistical inference methods have been widely applied to gain insight into which genes may influence the activities of others in a given omics data set, however, they do not provide information on the underlying mechanisms or whether the interactions are direct or distal. Therefore, interpretation of such a multitude of data requires an intuitive pathway context for biological interpretation, with efforts undertaken in integrated analysis of omics data within genome-scale metabolic reconstructions (Greenbaum et al., 2003; Arakawa and Tomita, 2013; Hyduke et al., 2013). We attempted to access the key differences in metabolic function between mature and immature tissues by developing a metabolic reconstruction based on the *S. italica* genome to integrate our omics data.

The reconstruction characteristics of *S. italica* and the published C_4_ model plant reconstructions (based on C4GEM) are presented in Table 1.

**Table 1:** **General characteristics of the C_**4**_ plant metabolic reconstructions**.

**Elements**	**C_4_ model plants (C4GEM)**			
	***Setaria italica***	***Sorghum bicolor***	***Zea mays***	***Saccharum officinarum***
ORF-reaction association entries	9363	13,114	38,892	13,593
Unique genes (ORFs)	1860	3557	11,623	3881
Metabolites	1690	1755	1755	1755
Extracellular transporters	18	18	18	18
Transporters (intercellular-plasmodesmata)	11	11	11	11
Transporters (interorganelle)	83	83	83	83
Genome size	~423 Mb (*2n* = 18)	~730 Mb (*2n* = 20)	~2.4 Gb (*2n* = 20)	~10 Gb (*2n* = 115)

*S. italica* reconstruction holds the main features of a C_4_ model plant. Primary metabolic functions were tested by gap filing and by manual curation by homology mapping to C4GEM. Although the C_4_ model plants have similar metabolic network topology, with the C_4_ traits and metabolic function of the typical C4 subtypes, the *S. italica* reconstruction has less gene-reaction associations and metabolites, compared to *Sorghum, Z. mays*, and sugarcane reconstructions because of its smaller genome.

In this study, the *S. italica* reconstruction platform was used to map multi-omics data so as to access to the overall metabolic contrast in young and mature tissues (complete mapping is presented in the supplementary file; Table S5). The same reconstruction can be refined and used to derive metabolic models. During modeling implementation, omics data, tissue biomass composition, and plant growth can be integrated as model-constraints to perform flux analysis. Here, the reconstruction was used for annotation and omics mapping and not used for modeling purpose.

### mRNA expression

RNA-Seq reads were mapped to the *S. italica* genome. Based on RNA-Seq analysis, 29,423 genes are estimated to be expressed in mature and immature tissues, which correspond to ~83% coverage of the protein coding genes of *S. italica* genome (Table 2). To complement this analysis, we used the *S.italica* metabolic reconstruction as a platform to assign genes to enzymatic function and to integrate omics datasets (complete mapping is presented in the supplementary file; Table S5). As indicated in Table 2, 15,788 genes (~54% the protein coding genes of *S. italica* genome) were differentially expressed in mature and immature tissues. Of these, 8361 genes (~31%) were mapped to the metabolic reconstruction and 5242 genes (~20%) were assigned to enzymatic reactions that were differentially expressed, indicating differences in metabolic regulation between mature and immature tissues.

**Table 2:** **Omics mapping of ***Setaria italic*****.

**Omics mapping**	
**TRANSCRIPTOME**	
Protein coding genes in *S. italica* genome^*^	35,424
*S. italica* mapped reads	29,423 (83%)
Genes differentially expressed in old and young tissues	15,788 (54%)
Genes expressed and mapped to the metabolic reconstruction	8361 (31%)
Genes differentially expressed and mapped to the reconstruction	5242 (20%)
**PROTEOME**	
Identified proteins	570
Protein differentially accumulated in mature and immature tissues	125
Protein mapped to the metabolic reconstruction	128
**METABOLOME (TARGET)**	
Identified metabolites	100
Metabolites differentially accumulated in mature and immature tissues	67
Metabolites mapped to the metabolic reconstruction	100

* *Phytozome v9 DB (Goodstein et al., 2012)*.

### Protein and metabolite abundances

To obtain a better understanding of changes in metabolic function with plant development, we assessed more than one “layer” of biological information to seek insights into the metabolic network. Proteins are the major components for building the cellular structure and they serve as catalytic enzymes in metabolic pathways. We were able to measure 570 proteins in immature and mature tissue samples, of which 125 (~22%) were significantly differentially abundant in mature and immature tissues (Table 2). The absolute quantitative measurement of low abundant proteins especially with complex samples is often hampered by technical constraints. Although protein coverage is low compared to the coverage of mRNA transcripts, many of the enzymes measured indicated different levels of abundances were found to that participate in central metabolic processes in mature and immature tissues.

The rate of enzymatic reactions is also regulated by concentrations of substrates and products (metabolites). Metabolites are the result of the interaction of the system's genome with its environment and reflect the response to physiological stimuli or genetic modification. We have used targeted metabolome analysis to measure metabolites of central carbon metabolism in order to capture their response in young and mature tissues of *S. italica*. One hundred metabolites were measured and mapped to the metabolic reconstruction, of which 67% were differentially abundant in both tissues.

### Overall analysis of central carbon metabolic pathways

The developed metabolic reconstruction was used to integrate our omics data, to capture the metabolic differences and similarities in young and mature tissues of *S. italica*, complementing the GO term analysis.

**Figure 2:**
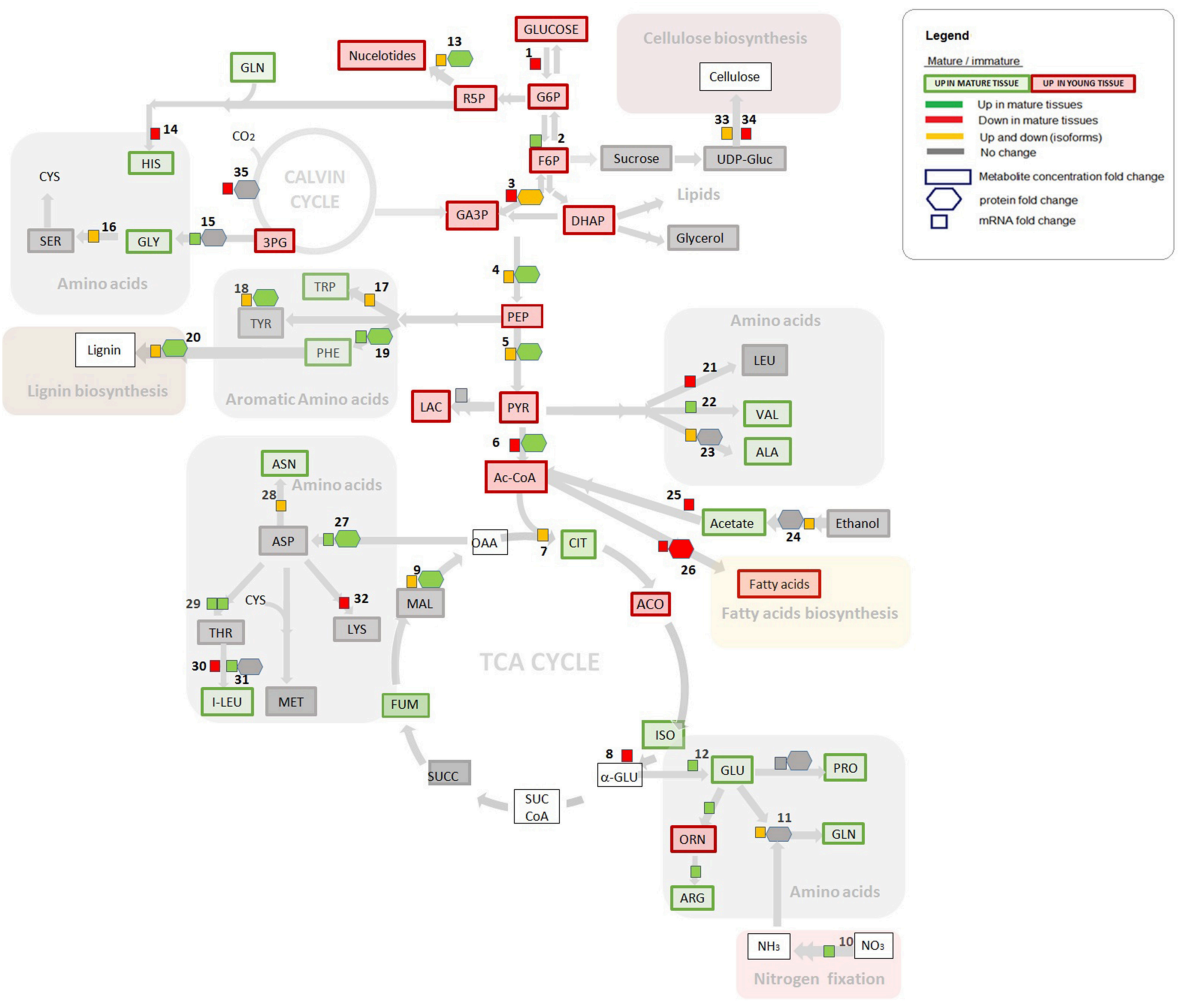
**Gene expression, proteome, and metabolome contrasts in young and immature tissues, assigned to the central carbon metabolism**. Gene expression and proteomics data are provided as log2FC (fold change) but for clarity of the figure only provided if either proteome or transcriptome changed by more than logFC of 0.5. Metabolite data is provided as concentration fold change. Omics data are highlighted in green, red, and yellow. Green: Increase in gene expression, protein accumulation, or in metabolite concentration in mature tissues compared to immature tissues. Red: Decrease in gene expression, in protein accumulation, or in metabolite concentration in mature tissues compared to immature tissues. Yellow: Up and down regulation of genes/enzymes that characterize isoforms in different tissues and organelles. Gray: No statistically significant difference between tissues. Numbers refer to enzymatic step reactions. Numbers (1–34) refers to enzymatic step reactions. 1, hexokinase; 2, glucose-6-phosphate isomerase; 3, glyceraldehyde 3-phosphate dehydrogenase (cytosolic and plastidial isoforms); 4, enolase (cytosolic and plastidial isoforms); 5, pyruvate kinase (cytosolic and plastidial isoforms); 6, pyruvate dehydrogenase complex; 7, citrate synthase (microchondrial and glyoxomal isoforms); 8, isocitrate dehydrogenase (mitochondrial); 9, malate dehydrohenase (mitochondrial, plastidic, glyoxysomal and cytosolic isoforms); 10, nitrate reductase; 11, glutamine synthetase; 12, glutamate synthase; 13, ribose 5-phosphate isomerase; 14, histidyl-tRNA synthetase; 15, 2-oxoglutarate aminotransferase; 16, glycine hydroxymethyltransferase; 17, tryptophan synthase; 18,transaminase; 19, aromatic amino acid aminotransferases; 20, peroxidase; 21, branched-chain amino acid aminotransferase; 22, valyl-tRNA synthetase; 23, aminoacyl-tRNA synthetase family protein; 23, alanine aminotransferase; 24, aldehyde dehydrogenase; 25, acetyl-CoA synthetase (acetate-CoA ligase); 26, fatty acid synthase (acyl-ACP synthases); 27, aspartate aminotransferase; 28, asparagine synthetase; 29, threonine synthase, threonyl-tRNA synthetase; 30, branched-chain amino acid aminotransferase; 31, isoleucyl-tRNA synthetase; 32, diaminopimelate decarboxylase; 33, glycosyl hydrolase family; 34, cellulose synthase; 35, Rubisco (carboxylation). Some steps were omitted for the sake of simplicity.

The most direct way to analyse omics data using a metabolic reconstruction and modeling platform is to compare omics measurements with the network topology or model predictions (Hyduke et al., 2013). The transcriptome and proteome data were processed and mapped into metabolic network topology for overall analysis and are presented in Table S5. Here, we have attempted to abridge our analysis by integrating the data in pathways of the central carbon metabolism, as depicted in Figure 2. Significant differences and similarities between mature and immature tissues of *S. italica* revealed by omics analysis are presented with particular attention to: fatty acids synthesis, biosynthesis of structural components like lignin, and cellulose, nitrogen fixation, amino acid synthesis, carbon fixation, and C_4_ metabolism.

### Fatty acids synthesis

Considering three levels of biological information revealed by transcriptome, proteome and metabolome data, an example of positive correlation is observed for the cellular components that participate in fatty acid synthesis (step 26, Figure 2). The metabolome analysis shows that the metabolites in glycolysis, which are the main metabolic building blocks for fatty acids (e.g., Acetyl- CoA, malony-CoA), and the saturated fatty acid products are significantly more abundant in young tissues (highlighted in red, Figure 2), in comparison to the correspondent levels in mature tissues. Interestingly, increased levels of unsaturated acids like omega 3, omega 7, and omega 9 are observed in mature tissues (Table S4). These mono and poly unsaturated fatty acids are produced from palmitic acid. According to our data, the saturated fatty acids are produced in young tissues, serving as the building blocks to produce the unsaturated fatty acids during the cell maturation.

In other plants, early studies have shown that the composition of fatty acids is under genetic control (Poneleit and Alexande, 1965), and their accumulation is influenced by environment, such as light, temperature (Dybing and Zimmerman, 1966), and also degree of maturity (Narayan and Joshi, 1971; Dasgupta and Friend, 1973). Other studies have performed comparative fatty acids analyses indicating higher levels of fatty acids in mature leaf tissues, when compared to young leaf tissues of other plant species (Chu and Tso, 1968; Sayanova et al., 1999).

#### Structural components: cellulose and lignin

In addition to generating gene expression profiles for mature and immature tissues, we mapped the differentially expressed transcripts to cellulose and lignin metabolic pathways in order to capture gene regulation over the structural biomass components in the two different developmental stages.

**Figure 3:**
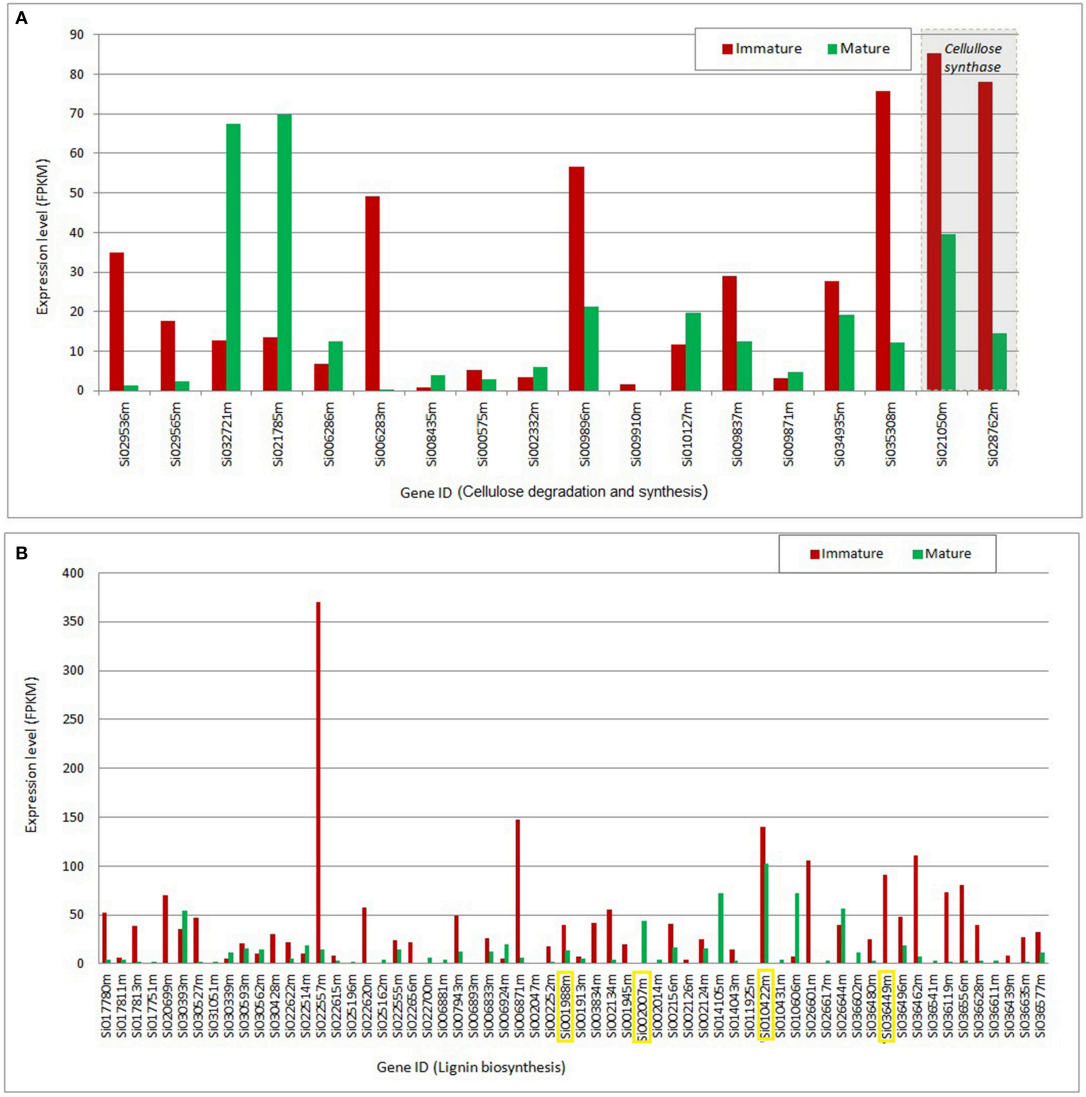
**Gene expression mapped to cellulose and lignin pathways. (A)** Gene expression in mature and immature tissues mapped to cellulose degradation (hydrolase family protein; EC 3.2.1.21) and synthesis (cellulose synthase; EC 2.4.1.12) as gene products. **(B)** Gene expression in mature and immature tissues mapped to lignin biosynthesis with peroxidases (EC 1.11.1.7) as gene products: Transcripts highlighted in yellow indicate that a corresponding protein product was detected by proteome analysis. FPKM, Fragments per kilobase of exon per million reads mapped.

Figure 3 presents the expression level of transcripts mapped to the synthesis and degradation of structural biomass components (cellulose and lignin) that are the most differentially regulated between mature and immature tissues.

Differentially expressed transcripts assigned to the degradation and synthesis of cellulose are presented in Figure 3A. This group is represented by two subsets of transcripts: One set with greater expression in immature, and another set preferentially expressed in mature tissues. This analysis indicates a coordinated regulatory function that may affect the flux through the cellulose synthesis and degradation pathways in an orchestrated manner throughout plant development. The transcripts mapped to cellulose synthesis (Si021050m and Si028762m) show the greatest expression level; two to five-folds increase in immature tissues, compared to their expression levels in mature tissues. One of the requirements for robust cellulose synthesis is the supply of its substrate UDP-glucose. Plasma membrane- associated sucrose synthase fulfills this function by catalyzing the formation of UDP-glucose from sucrose (Haigler et al., 2001). According to our metabolome analysis, UDP-glucose and sucrose pools are unchanged in mature and immature tissues of *S. italica* under the study conditions, suggesting that the cellulose changes over the plant development are not controlled by its substrates, but are most probably controlled at the transcriptional level.

Figure 3B presents the set of transcripts with significant differential expression in mature and immature tissues that were functionally assigned to lignin biosynthesis. For a few number of transcripts (highlighted in yellow, Figure 3B) we identified the correspondent protein abundance, which were higher in mature tissues (Figure 2, step 20). Overall, the data indicate higher expression in young tissues, suggesting up regulation of metabolic activity toward the cell wall lignification process, compared to the down-regulation in the mature stage of development, where the tissues may reach a metabolic plateau for the synthesis of cell structural components (like cellulose, lignin and hemi-cellulose).

Lignin is a significant structural biomass component, formed by a large group of aromatic polymers that are deposited predominantly in the walls of secondarily thickened cells, making them rigid and impermeable during tissue maturation (Vanholme et al., 2010). Cell wall lignification successively involves (i) the biosynthesis of monolignols in the cytosol, (ii) translocation of the monolignols to the cell wall and its preformed polysaccharide matrix, and (iii) oxidative polymerization of the monolignols to form the lignin polymer (Tobimatsu et al., 2013). Although it is still not clear how this process occurs, regardless of cell type undergoing lignification, carbon allocation to the different monolignol pools is apparently determined by a combination of phenylalanine availability and cinnamate-4-hydroxylase/“p-coumarate-3-hydroxylase” (C4H/C3H) activities, as revealed by transcriptional and metabolic profiling (Anterola and Lewis, 2002).

Based on our multi-omics analysis, phenylalanine; which is one of the precursors for lignin synthesis, is more abundant in mature tissues of *S. italica*. Moreover, the mRNA transcripts and the protein abundance (assigned to aromatic amino acid aminotransferases) are positively correlated to phenylalanine abundance (Figure 2, step 19). These data indicate that the synthesis of phenylalanine is controlled at the transcriptional and protein level and that the pool of this amino acid is increased over the tissue maturation process. Although the mRNA transcripts assigned to lignin synthesis are up-regulated in young tissues (Figure 3B), the lignification process throughout plant development is most probably controlled by the pool of available phenylalanine, which increases during tissue maturation.

It is known that the relative abundance of structural biomass components varies depending on tissue type, age, and environmental/biological condition of plant tissue (Campbell and Sederoff, 1996). Recently, the cell wall carbohydrates composition of Setaria was compared with other crop species (sorghum, switchgrass, and maize) at two developmental stages; (a) metabolically active young tissues and (b) metabolically plateaued; mature tissues (Petti et al., 2013). In this work, a consistent proportional decrease of cellulose in aerial tissues over the tissue maturation is reported. Consistently, this study shows that insoluble lignin content increased significantly between the immature and mature aerial tissue samples of the analyzed Panicoidae grasses. Our comparative omics analyses complement these findings. Altogether, these evidences indicate that the metabolic activity toward synthesis of the structural biomass components are most probably controlled at the transcriptional and protein level for cellulose synthesis and the lignification process is most probably limited by the pool of phenylalanine in the tissues.

#### Nitrogen fixation and amino acids synthesis

Overall, our data show that there is an increased in the amino acids pool in mature tissues (Figure 2) with a positive correlation for the mRNA transcripts assigned to nitrogen fixation (Figure 2, step10). Interestingly, out of the nine essential amino-acids, six are more abundant in mature tissues of the Setaria model plant (i.e., phenylalanine, valine, tryptophan, histidine, leucine, and isoleucine). Because of the interest in increasing the levels of essential amino acids in C_4_ crop plants (Ufaz and Galili, 2008), genetic strategies should be developed considering carbon partitioning, tissue-specificity and the ideal developmental stage, in order to achieve the best metabolic capacity to synthesize these essential amino acids in crop plants for the human/animal diet.

#### Isoforms

Many of the mRNA transcripts and the corresponding protein abundances were mapped to isoforms, indicating up or down regulation of the genes and enzyme products in the tissue samples (highlighted in yellow, Figure 2). Our data show no significant changes in the level of proteins over the plant development for the following group of isoforms: glutamine synthetase, 2-oxoglutarate aminotransferase, alanine aminotransferase, and aldehyde dehydrogenase (as shown in steps 11, 15, 23, 24; Figure 2). These data suggest that these step reactions are not regulated by the level of enzyme, but are most probably regulated at the transcriptional or post-transcriptional level during plant development.

In our study, the Setaria plants were growth under identical conditions and the differences highlighted here are related to the plant development (mature and immature tissues). Other studies show the regulation of isoforms in response to different plant treatments. For example, it has been reported that the regulation of glutamine synthetase isoforms is organ specific and occurred at transcriptional level in rice cultivars under drought tolerance treatments (Singh and Ghosh, 2013). In other work, evidence has been provided that the gene expression and the activity of alanine aminotransferase in soybean roots under hypoxic conditions varies depending on the nitrogen source that is supplied to the plants with ${\text{NH}}_{4}^{+}$ inducing alanine aminotransferase activity more than NO_3_ (Rocha et al., 2010).

### mRNA and protein abundances assigned to C_4_ metabolic pathways

**Figure 4:**
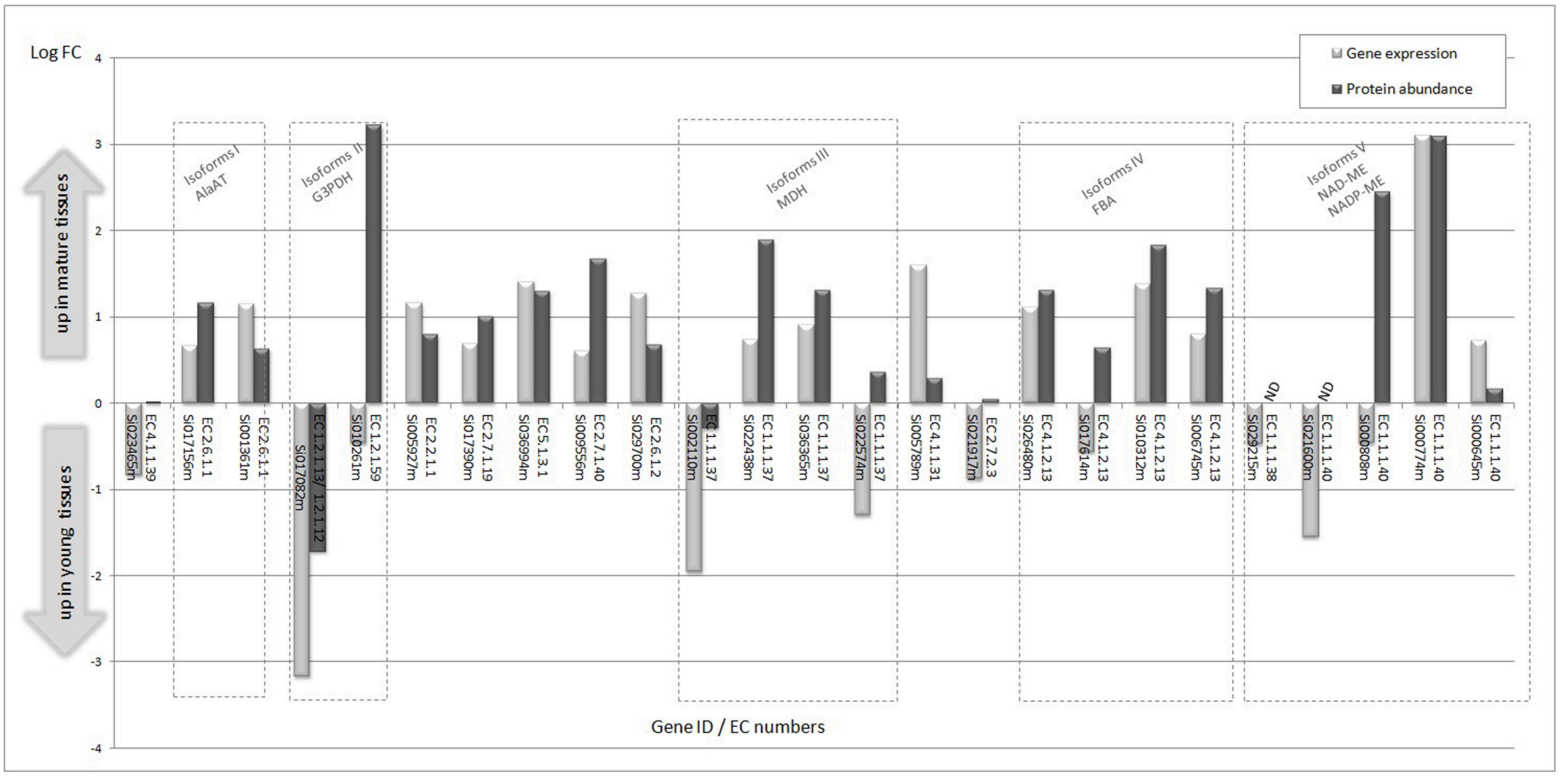
**Significant differential gene expression and corresponding protein abundance in young and immature tissues assigned to C_**4**_ metabolic pathways**. Isoforms are grouped in dashed rectangles. EC 4.1.1.39, Ribulose bisphosphate carboxylase small chain 1A/Rubisco small subunit 1A (RBCS-1A) (ATS1A); EC 2.6.1.1, aspartate aminotransferase; EC 1.2.1.13/EC 1.2.1.12, glyceraldehyde 3-phosphate dehydrogenase; EC 1.2.1.59, glyceraldehyde 3-phosphate dehydrogenase; EC 2.2.1.1, transketolase; EC 2.7.1.19, phosphoribulokinase; EC 5.1.3.1, ribulose-phosphate 3-epimerase; EC 2.7.1.40, pyruvate kinase; EC 2.6.1.2, alanine aminotransferase; EC 1.1.1.37, malate dehydrogenase; EC 4.1.1.31, phosphoenolpyruvate carboxylase; EC 2.7.2.3, phosphoglycerate kinase; EC 4.1.2.13, fructose-bisphosphate aldolase.

By performing multi-omics analysis, we have assessed the level of mRNA expression and the corresponding protein abundance in mature and immature tissues of enzymes and isoforms assigned to C_4_ metabolic pathways (Figure 4). In this group, a positive correlation of gene expression and protein abundance is observed, except for some of the isoforms (e.g., glyceraldehyde 3-phosphate dehydrogenase, malate dehydrogenase, and malic enzyme isoforms). The data indicate that most of the genes and the correspondingt proteins are up-regulated in mature tissues. Interestingly, higher expression of mRNA transcripts assigned to Rubisco carboxylase is observed in young tissues, but its corresponding levels of protein are unchanged in the tissue samples. Regulatory effects on Rubisco and PEPC are discussed in the carbon fixation session.

#### Transcripts assigned to malic enzyme isoforms are up-regulated during plant development

**Figure 5:**
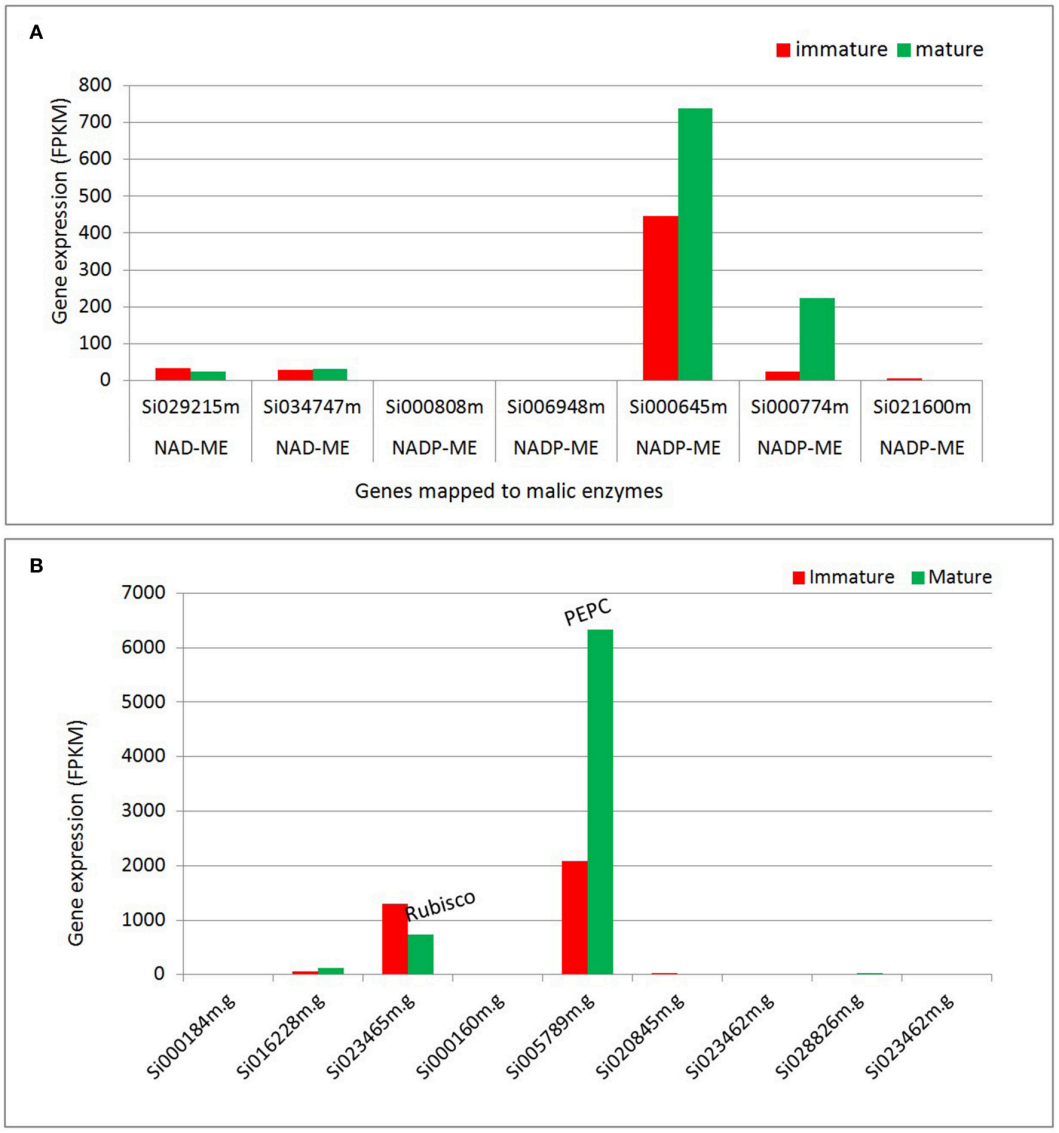
**Differential expression of transcripts assigned to enzymes that participate in carbon fixation. (A)** Transcripts assigned to malic enzymes. **(B)** Transcripts assigned to Rubisco and PEPC. NADP-ME, NADP malic enzyme; NAD-ME, NAD malic enzyme; Rubisco, Ribulose-1,5-bisphosphate carboxylase/oxygenase, PEPC, Phosphoenolpyruvate carboxylase.

Our transcriptome analysis reveals significant expression of transcripts assigned to malic enzyme isoforms (NADP-ME and NAD-ME; Figure 5A, Table S6). The transcripts assigned to NADP-ME show significant expression in both tissues, of which Si000645 shows the highest expression level; about 30-fold higher compare to the two NAD-ME isoforms (Si034747m and Si029215m). Overall, the two highly expressed NADP-ME isoforms (Si000645m and Si000774m) show higher expression levels in mature tissues (1.6 and 8.6-fold).

#### Carbon fixation

All C_4_ species operate on the same basic theme of pumping CO_2_ via C_4_ acids from M tissue, where phosphoenolpyruvate carboxylase (PEPC) activity is enhanced, to the BS layer, where Rubisco is localized and C_4_ acids are decarboxylated (Hatch, 1971, 2002; Hatch and Kagawa, 1976). The transcriptome analysis reveals the differential expression of transcripts mapped to enzymes that participate in carbon fixation (Figure 5B). The data indicates that Rubisco (carboxylase) and phosphoenol pyruvate carboxylase (PEPC) show the most significant levels of gene expression among the transcripts mapped to carbon fixation pathways. The analysis suggests that at the transcription level, PEPC and NADP-ME are up-regulated during plant development, but Rubisco is down regulated suggesting a possible switch from C3 to C4 type pattern with development (Figure 5). Other studies indicate that Rubisco transcription is suppressed in mesophyll due to poor mRNA stability in mature cells, but not in bundle sheath (Patel and Berry, 2008). The decrease in Rubisco transcript during development does not necessarily demonstrate a shift from C_3_ to C_4_ by itself, particularly as protein levels do not change. Nevertheless, it may reflect a shift from more general expression of Rubisco transcript in immature cells (a C_3_ type pattern) to specific expression of Rubisco in mature bundle sheath cells (a C_4_ pattern of expression). Therefore, in early development Rubisco transcription in mesophyll and bundle sheath may be similar.

**Figure 6:**
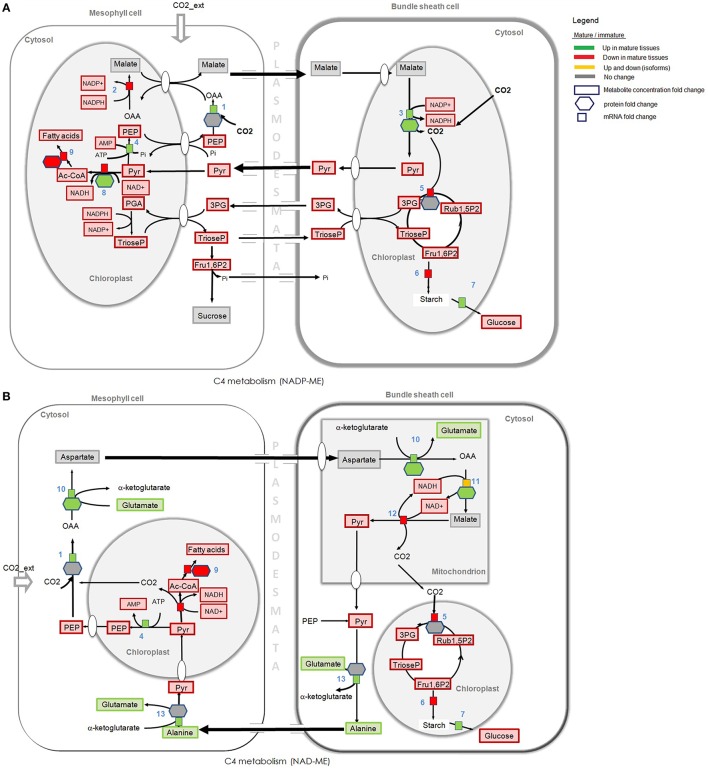
**Omics and C_**4**_ pathway analysis of two subtypes distinguished according to the decarboxylating enzyme in mature and immature tissues. (A)** NADP-ME, NADP requiring malic enzyme. **(B)** NAD-ME, NAD requiring malic enzyme. Gene expression and proteomics data are provided as log_2_FC but for clarity of the figure only provided if either proteome or transcriptome changed by more than logFC of 0.5. Metabolite data is provided as concentration fold change. Omics data are highlighted in green, red, and yellow. Green: Increase in gene expression, protein accumulation, or in metabolite concentration in mature tissues compared to immature tissues. Red: Decrease in gene expression, in protein accumulation, or in metabolite concentration in mature tissues compared to immature tissues. Yellow: Up and down regulation of genes/enzymes that characterize isoforms in different tissues and organelles. Gray: no statistically significant difference between tissues. Numbers refer to enzymes. (1) PEP carboxylase, (2) NADP-malate dehydrogenase, (3) NADP-malic enzyme, (4) Pyruvate-Pi dikinase, (5) Rubisco (carboxylation), (6) glucose-1-phosphate adenylyltransferase, (7) alpha /beta amylase, (8) pyruvate dehydrogenase complex (dihydrolipoamide S-acetyltransferase), (9) fatty acid synthases (saturated fatty acids), (10) aspartate aminotrasferase, (11) NAD-malate dehydrogenase, (12) NAD-malic enzyme, (13) alanine amino transferase. Some steps were omitted for the sake of simplicity.

Protein levels of PEPC and Rubisco did not change significantly between immature and mature phytomers (Figure 6, step 1 and 5). Previous studies show that Rubisco is strongly regulated at the protein level, which allows a lot of flexibility with respect to its enzyme activity. Majeran et al. (2010) observed that the Calvin-Benson cycle and the C_4_ shuttle increased substantially along the developmental gradient of a maize leaf, predominantly after cell elongation, cell wall deposition and plastid maturation were complete (Majeran et al., 2010). Wang L. et al. also saw this same increase in Calvin Benson cycle enzymes and C_4_ enzymes with respect to development (Wang L. et al., 2014). Additionally, Pick et al. (2011) observed that carbon assimilation rates steadily increase along the maturation gradient of the maize leaf, however, oxygen sensitivity of photosynthesis did not change along the leaf gradient suggesting a gradual sink-to-source transition without a distinct intermediary C3 stage (Pick et al., 2011).

Rubisco genes are known to be highly regulated; in many plants their expression is modulated by light (Lai et al., 2002), development (Hensel et al., 1993), and cell type (Sheen, 1999). Evidences show that C_4_ PEPC is regulated in response to diurnal fluctuations by a regulatory phosphorylation cycle (Bakrim et al., 1993). Although the multi-omcs data highlighted overall differences and similarities in mature and immature tissues, more studies in Setaria model plants are necessary to understand the mechanisms underlying these observations.

#### C_4_ photosynthesis and subtypes prints

C_4_ photosynthesis calls for metabolic compartmentation, which is in turn linked to specialized anatomy. Unlike C_3_ plants, where photosynthetic CO_2_ fixation proceeds in a single tissue, the mesophyll (M), in C_4_ plants, this process is distributed between mesophyll (M), and bundle sheath (BS) cells (Jensen, 1983; Hatch, 2002). C_4_ plants exhibit substantial variation in how they accomplish CO_2_ concentration. Traditionally, most of the C_4_ plants are classified into three subtypes (NADP-malic enzyme (ME), NAD-ME, or phosphoenolpyruvate carboxykinase (PEPCK) subtypes), according to their major decarboxylation enzyme (Hatch and Kagawa, 1976).

Although an overall analysis reveals that transcripts assigned to NADP-ME presented the highest level of expression (Figure 6A) in *S. italica* tissue samples, the transcripts mapped to NAD-ME also showed significant expression (see Table S5), suggesting some level of NAD-ME metabolic activity during carbon fixation. We have combined our transcriptome with proteome and metabolome analyses to investigate the pool of the C_4_ metabolic traits in young and mature tissues of the Setaria model plant (Table S4).

Figure 5 presents the multi-omics data mapped to C_4_ metabolism of two C_4_ subtypes (NADP-ME and NAD-ME). Green, red, and yellow colors represent significant differences in the total pool of the cellular components (mRNA, proteins, or metabolites) in mature and young tissues. Gray colors represent cellular components that show no significant differences in both tissues.

Carbon fixation in a NADP-ME subtype, the decarboxylation of malate (Figure 6A, step 3) takes place in the plastid of BS cells by NADP-ME enzyme. In a NAD-ME subtype, malate is decarboxylated in the mitochondria of BS cells (Figure 6B, step 12). Protein abundance and the expression level of transcripts mapped to NADP-ME (Figure 6A, step 3) are higher in mature tissues, but the concentration of metabolites that participate in C_4_ photosynthesis and carbon fixation is higher in immature tissues. The total pool of malate (the C_4_ metabolite that is decarboxilated by the NADP-ME in BS cells) is similarly high in mature and immature tissues but the pyruvate pool (the C_3_ metabolite that is translocated back to M cells via plasmodesmata) is higher in immature tissues. Aspartate is a compound normally considered as not present in the classical NADP-ME-type model, which is translocated from M to BS during C_4_ photosynthesis in NAD-ME subtypes. Interestingly, our data show that the pool of aspartate is also similarly high in mature and immature tissues (as the malate pool), and the alanine pool (the C_4_ metabolite that is translocated back to M cells in NAD-ME subtypes) is higher in mature tissues (see metabolome analysis in Table S3). These significant differences observed in the levels of mRNA transcripts, metabolites and proteins that participate in the NADP-ME and NAD-ME subtypes suggest that *S. italica* may use mixed decarboxilation modes of C_4_ photosynthetic pathways under different plant developmental stages. Omics analysis do not show significant gene expression or protein accumulation of PEPCK in young or mature tissues of *S. italic*.

Our measurements only capture the total pool of the C_4_ metabolic traits at transcriptional, metabolic and protein level. Although more biochemical studies at the organelles level are needed to confirm these findings, multiple evidences suggest that some flexibility in C_4_ photosynthetic pathways exists (Hatch, 1971; Chapman and Hatch, 1981; Jensen, 1983; Wang Y. et al., 2014). C_4_ photosynthesis has long been classified into three distinct subtypes, but early studies have shown coexistence of different C_4_ subtypes. Hatch (1971) demonstrated through ^14^C-labeling experiments in maize that radioactively labeled carbon provided as CO_2_ is mostly incorporated into malate, but also to a substantial degree into aspartate, a compound normally considered as not present in the classical NADP-ME-type model. A decade later, two other studies showed that isolated BS cells from maize can use aspartate and 2-oxoglutarate to produce CO_2_ (Chapman and Hatch, 1981) and that maize leaves contain sufficient activities of the aminotransferases to carry the required flux (Pick et al., 2011). Furthermore, a similar phenomenon has also been found in *Flaveria bidentis*, an NADP-ME dicot species (Meister et al., 1996). Considering these facts, it is highly likely that the so-called C_4_ subtypes actually coexist in C_4_ plants and such flexibility may be controlled by developmental and environmental cues. This is in keeping with the current view of C_4_ photosynthesis where a dynamic switching between all three subtypes of C_4_ photosynthesis occurs dependent on the relative energy and/or redox states of M and BS cells (Bellasio and Griffiths, 2014).

#### Correlation across multi-omics data

Overall, our analyses show little correlation between mRNA transcripts, the correspondent enzyme abundances and the metabolic product (when significant levels of protein and metabolite were detected) but a positive correlation is observed for biological components that participate in some pathways, such as in fatty acid synthesis and C_4_ metabolism.

Although one would hypothesize that the correlation between mRNA expression levels and protein abundance will be strong based on the central dogma of molecular genetics, support for this hypothesis from early experimental data is not immediately apparent. Most studies on microbes have either failed to find a significant correlation between protein and mRNA abundances (Gygi et al., 1999) or have observed only a weak correlation (Washburn et al., 2003; Nie et al., 2007; Zhang et al., 2010). It has been suggested that the discrepancy arises from several factors, including (i) protein regulation by post-translational modification, (ii) post transcriptional regulation of protein synthesis, (iii) differences in the half-lives of mRNA and proteins, (iv) possible functional requirement for protein binding, and (v) significant levels of experimental error. It has been generally accepted to think that for a group of genes showing a significant correlation between the reference mRNA and protein levels (Greenbaum et al., 2003), it is usually assumed that the cell has already put significant energy into dictating the final level of protein through tightly controlling the mRNA expression, and that there would then be minimal control at the protein level (steps 19 and 27 in Figure 2 may be an example of this case). In contrast, those genes that show minimal variation in their mRNA expression throughout the cell cycle are more likely to have little or no correlation with the final protein level (possible examples are shown in steps 6, 15, and 34; Figure 2). In this case, the plant cell would be controlling these genes at the translational and/or post-translational level, with the mRNA levels being somewhat independent of the final protein concentration (Greenbaum et al., 2003). These observations point toward the necessity for further target biochemical studies to investigate the underlying mechanisms that regulate the metabolic phenotype in C_4_ plants.

## Concluding remarks

By developing multi-omics protocols for systems analysis of the Setaria model plant we have demonstrated that integrated approaches can be combined with metabolic reconstruction platform to analyse the complexity of processes at different levels, and can provide insights into C_4_ metabolic traits. A metabolic reconstruction specific to Setaria along with an efficient transformation system might establish a more facile and higher throughput model for exploring metabolic traits for crop improvement and the potential of bioenergy grasses as biofactories. This is a critical first step in demonstrating the validity of using *Setaria* as a model C_4_ plant and is essential to realize any long-term benefits from the use of the platform to rapidly develop robust synthetic biology strategies, new plant biofactories and to explore the fundamentals of C_4_ metabolism.

## Author contributions

Dr. CD worked on the genome-scale reconstruction, multi-omics integration, overall pathway analysis, and manuscript preparation. Dr. CO did the proteome analysis. Dr. LG and Dr. JS worked on plant treatments and sample material preparation for the transcriptome analysis. Dr. MH, Dr. PC, and Dr. MP worked on metabolome analysis. Dr. RM did the plant treatments and sample preparation for the protein extraction. Dr. RP did the omics data processing. Prof. LN managed the project and designed the studies.

### Conflict of interest statement

The authors declare that the research was conducted in the absence of any commercial or financial relationships that could be construed as a potential conflict of interest.
