# Supplementary material for: Metabolic Reconstruction of Setaria italica: A Systems Biology Approach for Integrating Tissue-Specific Omics and Pathway Analysis of Bioenergy Grasses
Source: Front Plant Sci. 2016 Aug 10;7:1138. doi: 10.3389/fpls.2016.01138 (PMC4978736; doi:10.3389/fpls.2016.01138)
Supplement: Supplementary file 1 [file Table1.DOCX]

Table S1: Mobile phase gradient profile

| Time (min) | Eluent A (%) |
| --- | --- |
| 0 | 100 |
| 8 | 100 |
| 20 | 80 |
| 30 | 73 |
| 31 | 0 |
| 33 | 0 |
| 34 | 100 |
| 50 | 100 |
