# Supplementary material for: Metabolic Reconstruction of Setaria italica: A Systems Biology Approach for Integrating Tissue-Specific Omics and Pathway Analysis of Bioenergy Grasses
Source: Front Plant Sci. 2016 Aug 10;7:1138. doi: 10.3389/fpls.2016.01138 (PMC4978736; doi:10.3389/fpls.2016.01138)
Supplement: Supplementary file 2 [file Table2.DOCX]

Table S2: Analyte-dependent parameters for the transitions used in scheduled Multiple Reaction Monitoring data acquisition.

| **Analyte** | **Q1 Mass (Daltons)** | **Q3 Mass (Daltons)** | **RT (Minutes)** | **DP (Volts)** | **CE (Volts)** | **CXP (Volts)** |
| --- | --- | --- | --- | --- | --- | --- |
| Pyruvate | 87.02 | 43 | 12.5 | -45 | -12 | -1 |
| Lactate | 88.95 | 42.9 | 9.0 | -45 | -18 | -5 |
| Fumarate | 115.01 | 70.9 | 21.4 | -45 | -12 | -1 |
| Succinate | 117 | 73 | 19.0 | -45 | -16 | -3 |
| Oxaloacetate | 130.93 | 86.9 | 22.0 | -25 | -10 | -5 |
| Malate | 133 | 70.8 | 20.1 | -40 | -22 | -3 |
| Alpha-ketoglutarate | 144.95 | 100.8 | 20.9 | -40 | -12 | -5 |
| Phosphoenolpyruvate | 166.83 | 79 | 22.3 | -40 | -18 | -5 |
| Glyceraldehyde 3-phosphate | 168.84 | 97.1 | 10.2 | -40 | -10 | -5 |
| Dihydroxyacetone phosphate | 168.84 | 97 | 12.7 | -50 | -14 | -5 |
| Aconitate | 172.94 | 84.9 | 22.7 | -30 | -18 | -5 |
| 2 & 3-Phosphoglycerate | 184.91 | 97 | 21.7 | -50 | -20 | -5 |
| Isocitrate | 190.93 | 111.1 | 22.5 | -45 | -20 | -7 |
| Citrate | 190.96 | 110.9 | 22.5 | -50 | -18 | -7 |
| Ribose 5-phosphate | 228.94 | 96.9 | 9.9 | -65 | -18 | -5 |
| Ribulose 5-phosphate | 228.92 | 96.9 | 11.6 | -55 | -16 | -5 |
| Xylulose 5-phosphate | 228.93 | 97 | 11.4 | -50 | -18 | -5 |
| Glucose 1-phosphate | 259.02 | 78.8 | 10.9 | -65 | -48 | -3 |
| Glucose 6-phosphate | 258.89 | 96.7 | 8.9 | -75 | -22 | -5 |
| Fructose 1-phosphate | 259.02 | 96.8 | 10.5 | -55 | -22 | -5 |
| 6-Phosphogluconate | 274.93 | 97.1 | 21.5 | -60 | -24 | -5 |
| Cytidine monophosphate | 322.07 | 78.8 | 12.4 | -65 | -66 | -3 |
| Uridine monophosphate | 323.01 | 78.8 | 14.1 | -60 | -64 | -3 |
| Fructose 1,6-bisphosphate | 339.08 | 96.9 | 22.1 | -35 | -30 | -5 |
| Adenosine monophosphate | 346.02 | 78.6 | 16.5 | -70 | -62 | -3 |
| Guanosine monophosphate | 362.05 | 78.9 | 14.7 | -60 | -62 | -3 |
| Uridine diphosphate | 403.03 | 78.8 | 22.0 | -60 | -74 | -3 |
| Adenosine diphosphate | 426.07 | 78.8 | 22.5 | -85 | -74 | -3 |
| Guanosine diphosphate | 442.06 | 78.9 | 21.9 | -70 | -76 | -3 |
| Cytidine triphosphate | 481.97 | 158.6 | 29.3 | -75 | -36 | -11 |
| Uridine triphosphate | 483.06 | 158.8 | 30.4 | -65 | -42 | -7 |
| Adenosine triphosphate | 506.1 | 158.7 | 30.5 | -85 | -40 | -11 |
| Guanosine triphosphate | 522 | 158.7 | 30.0 | -80 | -42 | -11 |
| UDP glucose | 565.18 | 323 | 21.3 | -90 | -34 | -7 |
| UDP glucuronate | 579.14 | 79.1 | 29.7 | -90 | -108 | -1 |
| NAD | 662.25 | 540 | 13.6 | -50 | -20 | -9 |
| NADH | 664.2 | 78.8 | 22.7 | -110 | -98 | -1 |
| NADP | 742.2 | 620 | 21.7 | -45 | -24 | -11 |
| NADPH | 744.1 | 79.1 | 30.3 | -120 | -116 | -1 |
| Acetyl CoA | 808.17 | 79.1 | 32.3 | -125 | -54 | -5 |
| Cyclic AMP | 328.088 | 134 | 17.4 | -80 | -36 | -9 |
| Glyoxylate | 72.815 | 45.2 | 6.0 | -45 | -12 | -1 |
| Glycolate | 74.799 | 46.9 | 6.3 | -35 | -14 | -3 |
| Azidothymidine | 265.8 | 223.0 | 13.5 | -70 | -16 | -1 |
| Creatine phosphate | 209.738 | 78.8 | 19.7 | -35 | -16 | -3 |
| UDP N-acetylglucosamine | 605.857 | 78.7 | 21.4 | -95 | -106 | -1 |

Key: Q – quadrupole; RT – retention time; DP – declustering potential; CE – collision energy; CXP – collision cell exit potential
